# A Curious Critic’s Guide on Writing a Five-star Student Evaluation: Five Lessons Learned from Food Blogging

**DOI:** 10.7759/cureus.4372

**Published:** 2019-04-03

**Authors:** Xiao Chi Zhang, Meryl Abrams, Dimitrios Papanagnou

**Affiliations:** 1 Emergency Medicine, Thomas Jefferson University, Philadelphia, USA

**Keywords:** medical education, evaluation, feedback, faculty development

## Abstract

Timely feedback is critical in promoting learner self-reflection. When provided effectively, feedback can assist learners with the acquisition of new skills and knowledge in the ever-changing and complex landscape of healthcare. While the literature is replete on methods to provide feedback, faculty and supervisors receive little, if any, training on writing constructive feedback. Abbreviated comments (i.e., 'good job' or 'read more') provide little information on specific behaviors learners can change. As an avid food enthusiast and restaurant reviewer, I, too, am met with the challenge of writing a meaningful, constructive review (or evaluation) of a dining experience. To better assist clinical preceptors and supervisors with writing formative, constructive student evaluations, we have aggregated, reviewed, and adapted five lessons from writing food and restaurant reviews in writing a five-star student review.

## Editorial

Timely feedback is critical in promoting learner self-reflection. When provided effectively, feedback can assist learners with the acquisition of new skills and knowledge in the ever-changing and complex landscape of healthcare. The literature suggests that feedback be provided in a safe space, be based on direct observation, and incorporate specific suggestions for future improvement [1]. While the literature is replete on methods to provide feedback, faculty and supervisors receive little, if any, training on writing constructive feedback, in narrative format, as a means of formative assessment [2-3]. Abbreviated comments (i.e., ‘good job’ or ‘read more’) provide little information on specific behaviors learners can change to objectively improve their performance [4].

As an avid food enthusiast and restaurant reviewer, I, too, am met with the challenge of writing a meaningful, constructive review (or evaluation) of a dining experience [5]. While the stakeholders may differ, both narratives share a common literary syntax to accurately and succinctly convey aspects of the ‘observed encounter’. A well-written, structured evaluation has the ability to identify strengths and/or shed light on opportunities for improvement.

To better assist clinical preceptors and supervisors with writing formative, constructive student evaluations, we have aggregated, reviewed, and adapted five lessons from writing food and restaurant reviews. Through this parallel, our goals are to get evaluators to 1) reflect on their narrative feedback, 2) identify the extrinsic and intrinsic motivations of students, and 3) breakdown the critical components for writing a high-quality student ‘review’.

Lesson #1: Take [mental] pictures

Food Evaluation

**Figure 1:**
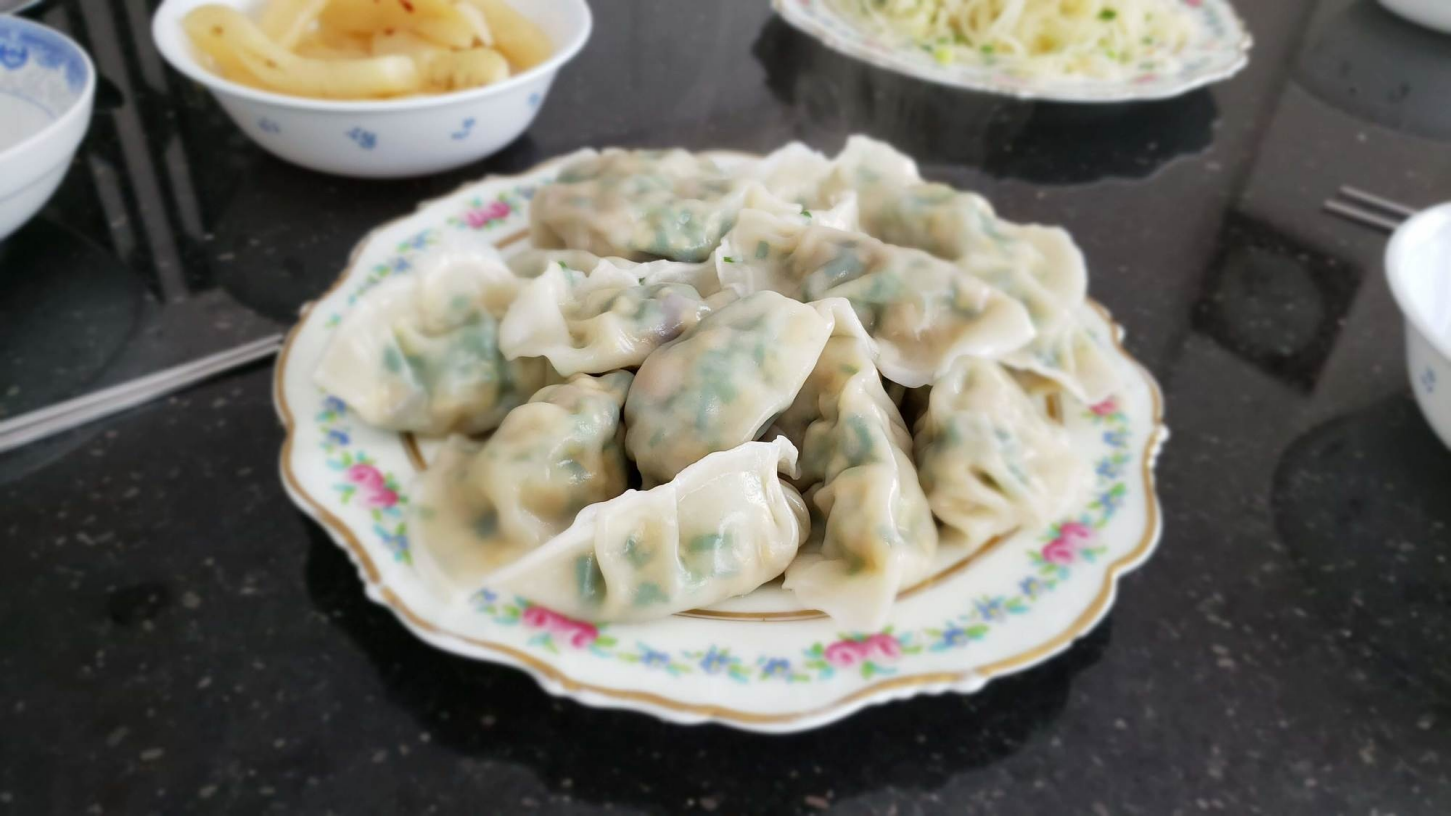
Steamed pork and chive dumplings Photographs taken by the primary author with permission to distribute

**Figure 2:**
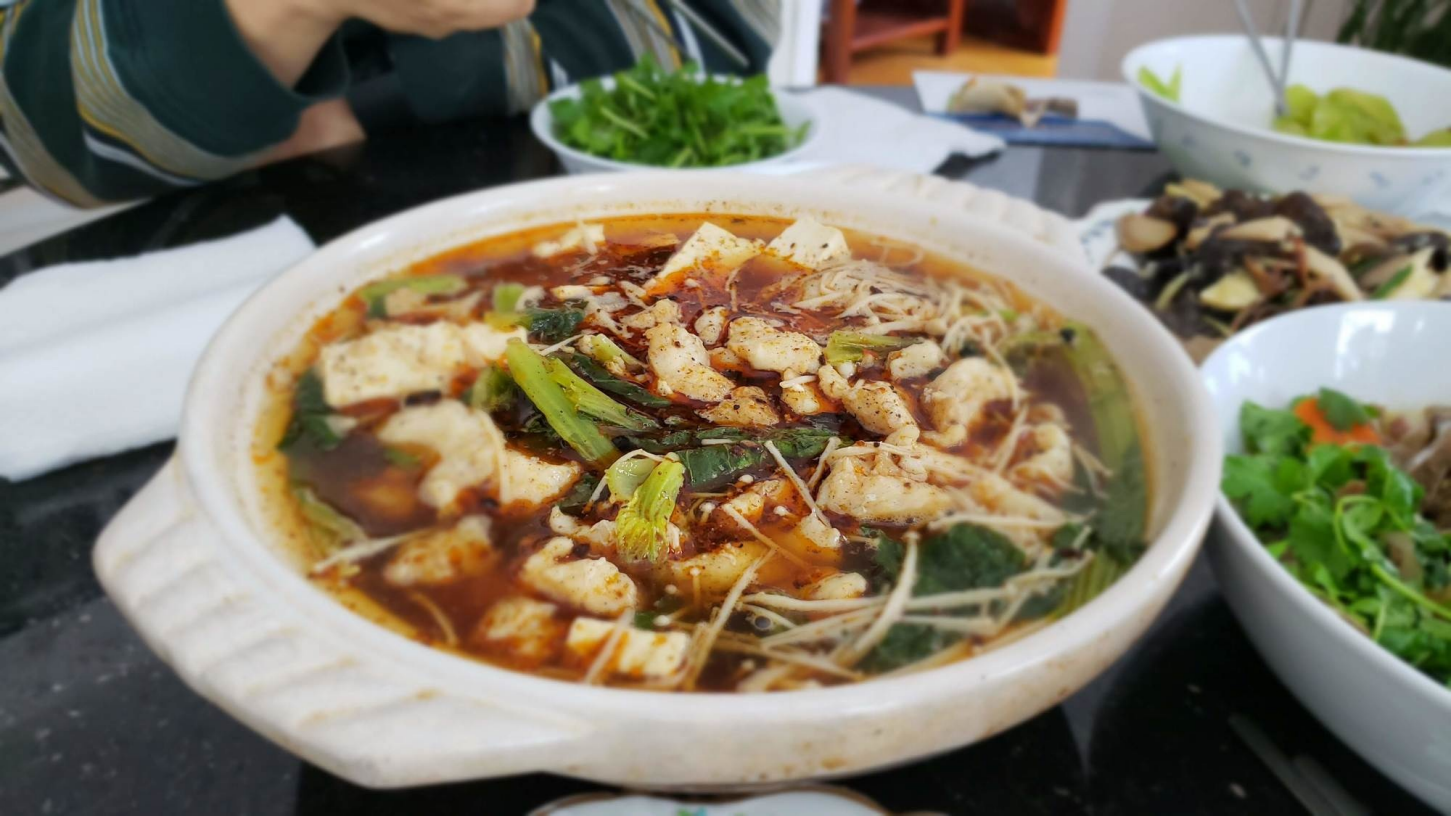
Spicy fresh-water bass and tofu stew in stone pot Photographs taken by primary author, with permission to distribute

Food enthusiasts typically choose to photograph their plated meals through candid snapshots to capture both the presentation and the artistic décor. While this practice may prompt an occasional odd glance or sneer, capturing an image of the culinary experience allows one to easily access a food-related memory objectively and with pinpoint accuracy. Examples for the food evaluation lesson #1 are displayed in Figures 1-2.

Clinical Evaluation

As clinical educators, we must constantly juggle patient stabilization, clinical teaching, learner supervision, and providing trainees with real-time feedback. While specific patients may invoke powerful memories of a clinical encounter, remembering the details surrounding a student’s performance after a hectic day is challenging and unreliable. Such retrieval, however, may be effectively facilitated through taking mental pictures of a student’s performance, simply by empowering the learners themselves to recall their own clinical experience for you. As an evaluator, you can ask the student to send you a formal evaluation request along with the following information: 1) the student’s professional photo; 2) his/her patient encounters of the day; 3) learning points; 4) areas of strength; and 5) ways to improve for the next day. This allows the learner to actively recapitulate their clinical experience while invoking the evaluator’s memory of the events surrounding the shift.

**Figure 3:**
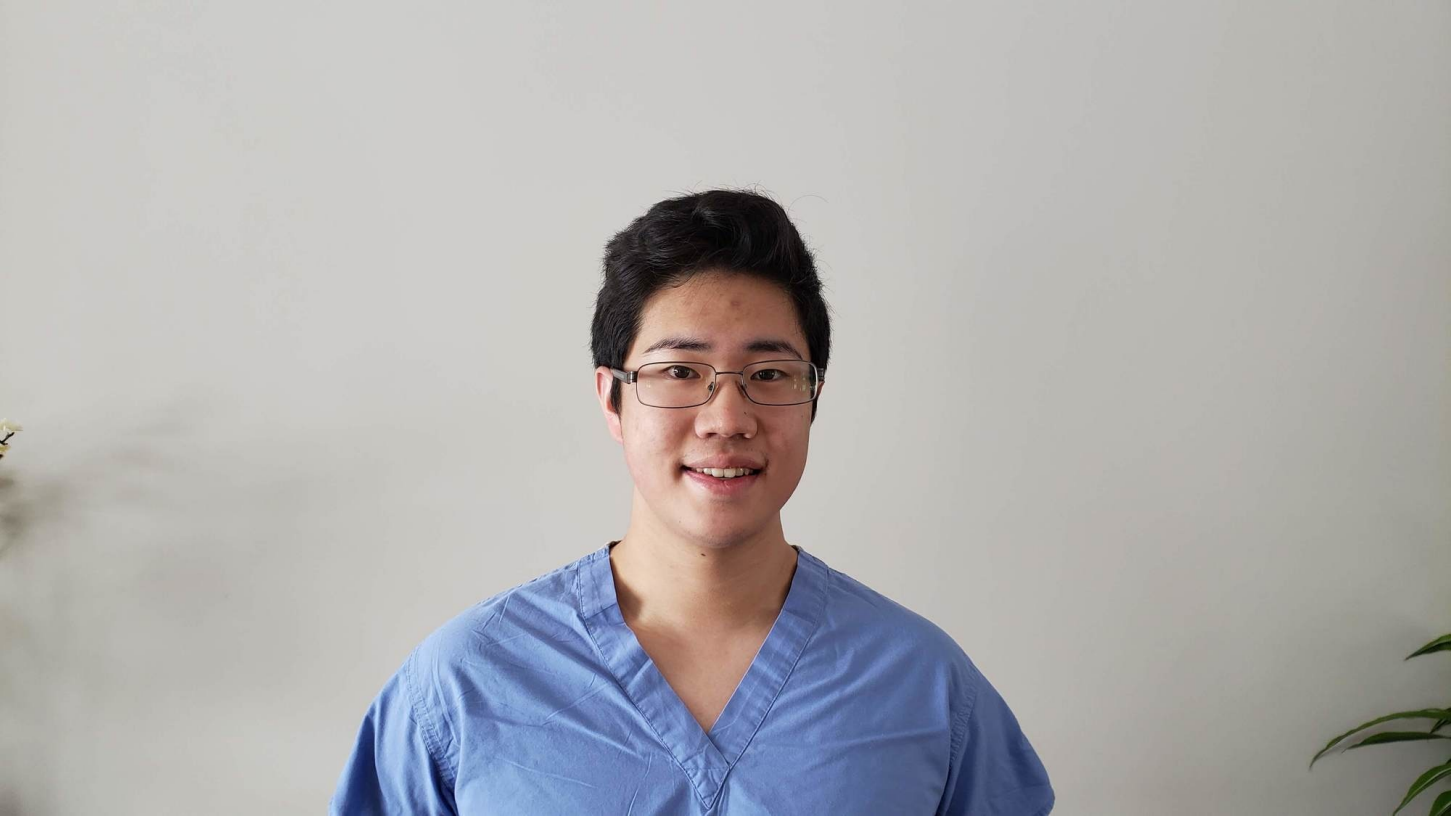
Student Z Photograph Photographs provided by the primary author

Clinical evaluation lesson #1 example: "Hi Dr. X, this is Student Z, the medical student you worked with on shift yesterday (Figure 3). I really enjoyed working with you. I learned a lot, including how to develop a differential diagnosis for acute abdominal pain and how to perform a hand laceration repair. I really appreciated your tips with calling consults. During the shift, I incorporated your tips when calling consults (i.e., for our patient with the Boxer’s fracture). We also took care of a young patient with intractable abdominal pain, requiring an interpreter phone. I also completed a complex laceration repair using simple interrupted techniques, and performed a bedside ultrasound to look for signs of an intrauterine pregnancy." *Disclaimer: Student Z is not an actual student.*

Lesson #2: Document every detail

Food Evaluation

Outstanding food reviews are trusted based on the reviewer’s ability to capture the culinary experience by painting a vivid, detailed image that stimulates the senses. Details are of utmost importance: the avant-garde décor, the artistically plated dishes, the attentive and friendly staff- collectively, all of these details frame the dining experience. Similarly, vivid descriptions of missed opportunities (or significant faux pas) provide an equally powerful red flag for future diners, to either pay closer attention to their restaurant experience or potentially avoid the restaurant.

Food evaluation lesson #2 example: "LiM’s House of Pancakes will transport you to a time when every ingredient is freshly picked and cooked from family tradition, passed on from generations of legendary chefs. The décor will remind you of a house kitchen with a few square tables that include edged hinges that allow it to convert to a larger circular table. There is no extraneous folk art or dining wear. Your ears will be tuned to the choreographed dances of the vegetables flipped high in the air with each wok toss, and your nose will be bathed with the aromatic scent of spices, garlic, and homemade fermented sauces from the nearby kitchen. Must get: homemade pork chive dumplings - they are served fresh from the boiling pot with an aroma that you can only get with in-season chives. The stickiness of the dumpling wrapper allows for easy picking, without the need for any utensils (chopsticks preferred)." *Disclaimer: LiM's House of Pancakes is a fictional restaurant*.

Clinical Evaluation

Clear and detailed documentation is as crucial when recording specific student behavior for the purpose of evaluation. A well-written, detailed student evaluation provides key information for both the student and his/her immediate supervisor (i.e., residency program director or student clerkship director). Specific student behaviors (i.e., proactively calling consults, effective suturing techniques, professionalism with staff, and appropriate documentation) provide formative markers to track their growth and note problem areas that require longitudinal monitoring. In addition to the noted strengths, pertinent negatives should also be documented.

Clinical evaluation lesson #2 example: "It truly gives me great pleasure to write a glowing and detailed report on Student Z’s performance on a very busy ED shift. Before our shift, I briefly worked with Z on a previous shift, when he evaluated a non-English speaking patient, incorporating translation only provided by the patient’s family. After we discussed the importance of using an interpreter phone, Z immediately incorporated this feedback during the current shift with all non-English-speaking patients, even though it would have been 'easier' to have the family interpret instead. Overall, Student Z is one of the most proactive, energetic, and enthusiastic students. He appreciates learning. His treatment plans improved and became more and more evidence-based over the course of the shift. Specifically, he followed-up on all of his patients and provided me with updates, as well as disposition and plans. He called consultants and admitted his own patients with minimal guidance. Student Z also shows great initiative. He performed a supervised bedside ultrasound on a pregnant female and was able to accurately demonstrate the views necessary for the transabdominal approach. Z was able to demonstrate an above-expected understanding of ECGs compared to many of his peers. I also observed him perform a complex laceration repair during the same shift with great dexterity."

Lesson #3: Use a rubric

*Food Evaluation* 

A five-star review, the pinnacle review for an outstanding culinary experience, provides concise and measurable outcomes that directly link to individual elements on a specific rubric. While scoring methodology (i.e., Likert scale, percentage, letter grade) may differ among critics, the final score rarely relies on taste alone. Texture, plating, aesthetics of both the plate and the establishment, cost, service, and establishment ambience all play a role in the overall evaluation.

Food evaluation lesson #3 example: "Food: 5 stars, Service: 4 stars, Cost: 3 stars, Ambience: 2 stars."

Clinical Evaluation

Similar to a food critic’s rubric, the United States medical students are evaluated according to a set of core competencies before graduating from medical school. The Association of American Medical Colleges (AAMC) lists 15 core competencies based on four major categories: interpersonal, intrapersonal, thinking and reasoning, and science. Similarly, the Accreditation Council for Graduate Medical Education (ACGME) uses a similar set of six core competencies to longitudinally evaluate the resident physician: patient care, medical knowledge, practice-based learning and improvement, interpersonal and communication skills, professionalism, and systems-based practice. Evaluators should adhere to the level of competence within each gradable competency to minimize subjective grading and ensure a consistent comparison between the student and her peers.

Clinical evaluation lesson #3 example: Table 1

**Table 1:** Sample rubric for practice-based learning and improvement

Practice-Based Learning and Improvement			
Below Expected	Expected	Above Expected	Not Applicable
Accepts and incorporates feedback. Limited understanding of evidence-based medicine principles and their application to patient care. Limited application of electronic medical resources	Describes the basic principles of evidence-based medicine. Actively seeks and incorporates feedback on shift. Can identify and appropriately use electronic medical resources	Performs patient follow-up. Effectively utilizes evidence-based medicine. Effectively utilizes electronic medical resources and weighs their reliability (i.e. Free Open Access Medical Education, online textbooks).	Not Applicable

Lesson #4: Leave room for improvement

Food Evaluation

It is rare for every dining experience to exceed all expectations. When there is a discrepancy between expectation and reality, the food critic must always acknowledge the chef’s [or owner’s intentions] prior to submitting an unfair review of the establishment. For example, a ‘hole-in-the-wall’ restaurant that specializes in serving freshly-steamed, traditional, Beijing dumplings can open a cultural portal to another part of the world; this establishment should not be expected to have the same luxury dining setting provided by a 3-starred Michelin hotel-restaurant. When appropriate, a well-written review should highlight specific examples of deficiencies, while offering the restaurant opportunities for improvement. Owners are now accountable for addressing these shortcomings. Should they choose, they now have the insights to reflect and address noted recommendations for future clients.

Food evaluation lesson #4 example: "This was an absolutely flawless, 11 out of 10 (!), culinary experience. LiM’s House of Pancakes faithfully brings authentic Beijing cuisine in an affordable and aesthetically pleasing manner. My only constructive feedback is that this establishment is CASH-only, and there were limited displays that illustrated this preferred payment manner. Also, for a restaurant named after their pancakes, they ran out of them by 2pm! I recommend the owners should prepare more of their flagship pancake dishes and place a larger and more visible banner on the storefront to alert customers of this before they sit down to the best meal of their life!"

Clinical Evaluation

Clinical rotations represent the steepest learning curves for many students in the health professions. Students are held accountable for their learning in the clinical environment. To this effect, clinical preceptors must provide students with constructive feedback that is purely based on direct observation following each encounter, as well as document performance through a formal evaluation that will later be reviewed by a clerkship or program director. Noted areas for improvement can be instrumental in providing targeted remediation and mentorship for struggling learners.

Clinical evaluation lesson #4 example: "When there is diagnostic uncertainty, please do not hesitate to look up potential risk-stratifying and decision making tools to better apply evidence-based recommendations and work-ups. Student Z’s initial presentations were a bit too long and unfocused with intermittent historical distractors. With direct feedback and guidance, however, his presentations became much more concise as the shift progressed." 

Lesson #5: Check-in before you check out

Food Evaluation

Food critics have different intrinsic and extrinsic motivations that compel them to write a review at the end of their dining experience. In today’s hectic life, time is sparingly short and always in demand. As a result, even the most well-intentioned and constructive reviews can be buried and forgotten without proactive reminders. Many review apps, such as Yelp (www.yelp.com), now enable end-users and reviewers to ‘check-in’ to the restaurant, which will send periodic reminders to submit an evaluation of the dining experience. ‘Check-in’ reminders can be helpful in collecting an otherwise forgotten review before the critic ‘checks-out’ of the experience.

Food evaluation lesson #5 example: Using digital apps to mark the moment you arrive (i.e. check-in) at a dining establishment to set up an automatic reminder to write a future review.

Clinical Evaluation

Unlike real-time feedback, written evaluations are challenging to complete at the end of a busy clinical shift, when both students and preceptors are trying to tie up the loose ends. It is quite common for students and preceptors to forget the evaluation. Institutions that utilize electronic evaluations often require learners to send- out individualized evaluation requests through a password-secured hospital server, making it difficult for evaluators to submit their reviews without student-driven prompts. Evaluators can navigate this by requesting the student to send an evaluation request at the beginning of the shift as a ‘check-in’ reminder. This ‘check-in’ serves as a reminder for both the evaluator to complete the evaluation, and for the student to acknowledge that his/her performance will be assessed during the shift.

Clinical evaluation lesson #5 example: Encourage your students to send you a shift evaluation request at the beginning of the clinical shift and set the priority to ‘high.’ For example: "Dear Dr. X, this is Student Z. Please do not forget to complete an evaluation after today’s shift. Thank you in advance."

In conclusion, constructive, narrative, written feedback is an integral part of the formative and summative evaluation in health professions education. While many educators may still feel uncomfortable and unprepared to deliver this type of feedback to their learners, we posit that incorporating insights from food blogging can alleviate writers' blocks and promote timely and characteristic student reviews. As teachers and evaluators, the written feedback is highly prized by all members of the medical education paradigm and has the potential of guiding the practice patterns of our future clinicians. We hope the five-step guide - 1) take a mental picture; 2) document details; 3) use a rubric; 4) leave room for improvement; and 5) checking-in - to developing a glowing student review will support evaluators as they aim to provide written, constructive feedback to their students.
